# ANMCO (Italian Association of Hospital Cardiologists) scientific statement: obesity in adults—an approach for cardiologists

**DOI:** 10.1007/s40519-023-01630-8

**Published:** 2024-01-02

**Authors:** Stefania Angela Di Fusco, Edoardo Mocini, Michele Massimo Gulizia, Domenico Gabrielli, Massimo Grimaldi, Fabrizio Oliva, Furio Colivicchi

**Affiliations:** 1 Emergency Department, Clinical and Rehabilitation Cardiology Unit, San Filippo Neri Hospital, ASL Roma 1, Rome, Italy; 2 Department of Experimental Medicine, Sapienza University, 00161 Rome, Italy; 3 Cardiology Department, Garibaldi Nesima Hospital, 95122 Catania, Italy; 4 Cardio-Thoracic-Vascular Department, San Camillo-Forlanini Hospital, Rome, Italy; 5 Department of Cardiology, General Regional Hospital “F. Miulli”, Acquaviva delle Fonti, 70021 Bari, Italy; 6 De Gasperis Cardio Center, Niguarda Hospital, 20162 Milan, Italy; 7 Heart Care Foundation, Florence, Italy

**Keywords:** Obesity, Energy homeostasis, BMI, Weight loss, Fat tissue, Cardiology, Adult

## Abstract

Obesity is a complex, chronic disease requiring a multidisciplinary approach to its management. In clinical practice, body mass index and waist-related measurements can be used for obesity screening. The estimated prevalence of obesity among adults worldwide is 12%. With the expected further increase in overall obesity prevalence, clinicians will increasingly be managing patients with obesity. Energy balance is regulated by a complex neurohumoral system that involves the central nervous system and circulating mediators, among which leptin is the most studied. The functioning of these systems is influenced by both genetic and environmental factors. Obesity generally occurs when a genetically predisposed individual lives in an obesogenic environment for a long period. Cardiologists are deeply involved in evaluating patients with obesity. Cardiovascular risk profile is one of the most important items to be quantified to understand the health risk due to obesity and the clinical benefit that a single patient can obtain with weight loss. At the individual level, appropriate patient involvement, the detection of potential obesity causes, and a multidisciplinary approach are tools that can improve clinical outcomes. In the near future, we will probably have new pharmacological tools at our disposal that will facilitate achieving and maintaining weight loss. However, pharmacological treatment alone cannot cure such a complex disease. The aim of this paper is to summarize some key points of this field, such as obesity definition and measurement tools, its epidemiology, the main mechanisms underlying energy homeostasis, health consequences of obesity with a focus on cardiovascular diseases and the obesity paradox.

*Level of evidence* V: report of expert committees.

## Introduction

Most physicians, upon encountering a patient with obesity, often attribute this condition to excessive food consumption and/or a sedentary lifestyle, perceived as resulting from a lack of knowledge about healthy living, or a lack of willpower to establish healthy habits. At the societal level, this simplistic approach is frequently associated with a moral judgment that connects obesity with a weakness of character or, more derogatorily, other negative traits. Such attitudes rapidly lead to social stigma. In healthcare settings, this stigma surrounding obesity can result in a lower quality of care [1]. Some cardiologists may not be aware of the underlying diseases or factors that could trigger or perpetuate this chronic and complex condition and may skip the diagnostic step to jump directly to the therapeutic phase by recommending weight loss. Occasionally, the patient is referred to a dietitian, nutritionist, or personal trainer as though obesity were not a chronic disease but simply the outcome of poor habits. However, these habits rely on many factors, not all of which are under the patient’s control. The achievement of weight loss often becomes the primary goal, eclipsing the importance of promoting overall health and understanding the complex, heterogeneous, multifactorial, and chronic nature of obesity as an epidemic. Understanding the underlying factors can aid in tailoring treatment, guiding patients, improving their understanding of the disease, increasing their commitment and self-efficacy, and enhancing their treatment adherence. Offering accurate advice also discourages patients from turning to commercial weight-loss programmes, which can be not only ineffective but also detrimental to physical and mental health. The aim of this article is to summarize what cardiologists need to know to effectively manage patients living with obesity, and to underscore the importance of a multidisciplinary approach. We discuss the primary pathophysiological mechanisms underlying obesity and obesity-related cardiovascular diseases. Moreover, this document focuses on measurement tools useful in identifying patients with obesity and assessing the cardiovascular risk associated with obesity.

## Definition and measurement tools

Obesity is defined as an abnormal or excessive fat accumulation that poses a health risk [2]. Visceral abdominal fat is more strongly associated with adverse health effects as compared to the amount of total body fat [3, 4].

Body Mass Index (BMI) is the most used obesity measure both in scientific literature and in clinical practice. This measure is obtained by dividing weight in kilograms by the square of height in meters [5]. BMI is a simple, easy, and inexpensive way to identify patients living with obesity. However, at the individual level, using BMI as the only tool for the diagnosis and staging of obesity presents important critical issues. BMI cannot distinguish body composition, it does not consider the location of adipose tissue in the body, so it may under- or over-estimate metabolic and cardiovascular risk related to the obesity.

In general, overall obesity and abdominal obesity are directly related [5]. However, it is possible to find patients with a BMI of less than 30 kg/m^2^ and abnormal visceral fat accumulation (normal-weight obesity) [5]. On the other hand, a person classified as affected by obesity only using BMI, could have a non-visceral fat distribution [5].

The simultaneous presence of excess fat and loss of lean body mass is defined as sarcopenic obesity. Recently, a joint ESPEN (the European Society for Clinical Nutrition and Metabolism) and EASO (the European Association for the Study of Obesity) international consensus proposed screening and diagnostic criteria that concern both body composition and functional and strength tests, inviting to consider the co-presence of the two pathologies as a separate pathology different from sarcopenia or obesity alone, due to the synergistic effect and the consequent increase in the risk of dysmetabolic pathologies [6].

Furthermore, BMI does not take into account different body composition age-related [e.g., due to the loss of fat-free mass (FFM) associated with ageing], sex- and ethnicity-related. Different cut offs should be used in patients belonging to populations with different characteristics [7]. As an example, lower BMI cut offs should be used to define overweight and obesity in Asian populations [8, 9]. In these populations overweight and obesity should start, respectively, at BMI 23 kg/m^2^ and 27.4 kg/m^2^, [9].

Subcutaneous and visceral adipose tissues have numerous anatomic and functional differences. Subcutaneous adipose tissue (SAT) accounts for approximately 80% of total fat, while visceral adipose tissue (VAT) accounts for 10–20% [10]. Blood from the VAT is drained directly to the liver via the portal vein system, whereas blood from the SAT is drained via the systemic venous system.

The quality and quantity of cytokines secreted appears to differ particularly with the increase in fat mass [11].

When VAT increases and exceeds a certain level, pathological processes begin. They are mediated by an increased quantity of cytokine secreted, a quantitative and qualitative change in immune system activity, fat hypertrophy, increased fibrosis, and impaired vascular function and structure [11–13]. All these tissue and functional changes are referred to as adiposopathy [12].

The increase in the accumulation of adipose tissue in the subcutaneous tissue is progressively associated with an increase in visceral adipose tissue with a non-linear and very individual relationship. The organs most involved are the liver, pancreas, epicardium and pericardium, and muscle tissue. In such cases, it is called ectopic fat, which must be considered equal to visceral fat in terms of cardiovascular risk [4].

Due to the above mentioned limits of BMI as a measure of body fat, several other measures have been proposed to better define the presence and distribution of adipose tissue (Table 1) [14, 15], including waist circumference (WC), waist-to-hip ratio (WHR), waist-to-height ratio (WHtR), the fat mass index (FMI), fat-free mass index (FFMI), fat mass body percentage (FM%), but none of these alone has been consistently found to be a better predictor for clinical purposes [16, 17].

**Table 1:** Weight categories and waist circumference cut-off values

Weight categories according to BMI in adults [8]		
	kg/m^2^	
Underweight	< 18.5	
Normal weight	≥ 18.5 and < 25	
Overweight	≥ 25 and < 30	
Obesity	> 30	
Class I	≥ 30 and < 35	
Class II	≥ 35 and < 40	
Class III	≥ 40	Also named severe, extreme, or morbid obesity

Waist circumference cut-off indicating increased health risk in various ethnicities [18, 19]		
	M (cm)	F (cm)
Caucasian Europid/United States/Mid-East Mediterranean^b^	102	88
Latino Central/South American^b^	94	90
Sub-Saharan African^a^	95	99
African^b^	80.5	81.5
Korean^b^	90	85
Chinese^a^	83	81

World Health Organization classification of body weight for Caucasian people. In people with an Asian, Chinese, Middle Eastern, Black African, or Afro-Caribbean family background, obesity classes 2 and 3 are usually identified by reducing the values used for Caucasian people by 2.5 kg/m^2^ because they are prone to central adiposity and their cardiometabolic risk occurs at a lower BMI. In these populations overweight and obesity start respectively at BMI 23 kg/m^2^ and 27.4 kg/m^2^ [8]

^a^Increased cardiovascular risk

^b^Greater increasing cardiovascular risk

Probably, WC is the simplest and most economical measure of abdominal obesity [18]. Of note, as well as for BMI, to define increased adiposity different waist circumference cutoff values have been proposed on the basis of sex and ethnicity [19, 20].

To obtain the most accurate measurement of VAT without resorting to complex instrumental examinations, the use of certain anthropometric measurements have been proposed and validated [21].

Proximal thigh circumference (PTC) and WC have been identified as proxy indicators of subcutaneous abdominal adipose tissue (SAAT) and the sum of SAAT and VAT, respectively. By subtracting the SAAT value from the waist circumference and correcting the result for age, sex, and BMI, a VAT value is obtained, which correlates with VAT measured with computed tomography (CT) [21]. This, along with magnetic resonance imaging (MRI) are currently gold standard methods to measure VAT.

The amount of adipose tissue in the human body can be detected using different invasive and non-invasive techniques. Non-invasive methods include hydrostatic weighing (densitometry—HW), air displacement plethysmography (ADP), bioelectrical impedance analysis (BIA), dual-energy X-ray absorptiometry (DXA), CT, MRI [14]. Table 2 presents some characteristics of these techniques.

**Table 2:** Body composition evaluation methods

	Ability to assess FM and FFM	Ability to assess fat regional distribution	Precision and accuracy in measuring VAT	Use of ionizing radiation
Circumferences	Yes	Yes	Low	No
Skinfold thickness	Yes	Yes	Low	No
Ultrasound	No	Yes	Low	No
Hydrostatic weighing densitometry	Yes	No	High	No
Air displacement plethysmography	Yes	No	High	No
Bioelectrical impedance analysis	Yes	No	Medium	No
Dual-energy X-ray absorptiometry	Yes	Partially	High	Yes
Computed Tomography	Yes	Yes	Gold standard	Yes
Magnetic Resonance Imaging	Yes	Yes	Gold standard	No

Most used methods to evaluate body composition

FM, fat mass; FFM, fat-free mass; VAT, visceral adipose tissue

In summary HW, ADP and BIA can give information on the overall body density and so can provide a measure of total body fat and lean tissue, but they cannot specify fat regional distributions. With this purpose, CT and MRI are the most accurate and precise techniques. They can detect and measure fat distribution, such as visceral, muscle, or liver fat. In some instances, a subject with normal BMI but elevated intra-abdominal fat could be differentiated from a high BMI subject with normal intra-abdominal fat, resulting in a more accurate cardiovascular risk assessment. Other details, if needed, can be obtained in Borga et al. [14].

Despite the limitation expressed above, an elevated BMI remains a simple, inexpensive, measure, associated with a poorer outcome and constitutes the basis for the World Health Organization (WHO) definition of overweight and obesity (see Table 3).

**Table 3:** Methods to assess body adiposity [14]

Measurement	Acronymous	Measured/calculated	Screening	Clinical setting: diagnosis/ management	Research Setting
Body mass, Kg	BM	Measured (Kg)	+	+	+
Body mass Index, Kg/m^2^	BMI	Weight in kilograms divided by height (h) in meters squared (m^2^)	+	+	+
Body fat mass, Kg	FM	Measured by instruments *(BIA–DEXA–MRI–CT)*		+	+
Body fat mass index, Kg/m^2^	FMI	FM/h^2^		+	+
Fat-free mass, Kg	FFM	Measured by instruments *(BIA–DEXA–MRI–CT); calculated: BM–FM*		+	+
Body fat percentage, %	%BF	FM*100/BM		+	+
Fat-free mass index, Kg/m^2^	FFMI	FFM/h^2^		+	+
Visceral adipose tissue, L, ml, Kg, gr, cm^2^	VAT	Measured or calculated by anthropometric measures, MRI and CT gold standard		+	+
Waist Circumference, cm	WC	Tape measure	+	+	+
Thigh Circumference, cm	TC	Tape measure			+
Neck Circumference, cm	NC	Tape measure			+
Hip circumference, cm	HC	Tape measure	+	+	
Total abdominal adipose tissue, cm^2^	TAAT	Measured by RMI			+
Subcutaneous adipose tissue, cm^2^, or as CT/MRI	SAT	Measured or calculated by anthropometric measures, MRI and CT gold standard			+
Subcutaneous abdominal adipose tissue, cm^2^, or as CT/MRI	SAAT	Measured or calculated by anthropometric measures, MRI and CT gold standard			+
Waist-to-hip ratio	WHR	Calculated (WC/HC)	+	+	+
Waist-to-height ratio	WHtR	Calculated (WC/h)	+	+	+
Waist-to-thigh ratio	WTR	Calculated (WC/TC)			+

h,  height in meters; L, litres; ml, millilitres; Kg, kilograms; gr, grams; BIA, bioelectrical impedance analysis; DXA, dual-energy X-ray absorptiometry; CT, Computer Tomography; MRI, Magnetic Resonance Imaging

A BMI between 25 and 29.9 identifies an overweight individual, a BMI of 30 or more indicates individuals with obesity [2].

In people with an Asian, Chinese, Middle Eastern, Black African, or Afro-Caribbean family background, obesity classes 2 and 3 are usually identified by reducing the values used for Caucasian people by 2.5 kg/m^2^, because they are prone to central adiposity and their cardiometabolic risk occurs at a lower BMI. In these populations overweight and obesity start, respectively, at BMI 23 kg/m^2^ and 27.4 kg/m^2^ [9]. Table 1 reports obesity classification based on BMI values and waist circumference cutoff [19].

### Take-home message

Although CT and MRI are the gold standard for adipose tissue quantification and localization these methods are infrequently used in clinical practice. BMI and waist-related measures (WC, WHtR, WHR) cannot completely replace them but, in a simple daily clinical purpose, can be used as excellent screening tools. They should be integrated, in individual cases, with additional tools for a better definition of individual patient body composition and related risk (DXA, BIA) [9, 15].

## Epidemiology

More than 50% and nearly 15% of adults in the Organisation for Economic Co-operation and Development (OECD) countries are, respectively, overweight or obese [22]. In Italy, 35% of the population is overweight and more than 9.8% is obese [23]. In high-income countries, such as the United States, nearly, 40% of adults met the BMI criteria for obesity in 2015–2016, with approximately 8% meeting criteria for grade 3 obesity (6.2% in males and 10.5 in females) [24].

In 2015, all over the world 107.7 million children and 603.7 million adults were living with obesity [25]. The overall prevalence of obesity was 5% among children and 12% among adults [26]. The expected growth is impressive. The United States, Mexico, and England are expected to have 47%, 39%, and 35% of the population obese, respectively [22]. A large increase in overweight and obesity is also evident in low- and middle-income countries [25].

### Take-home message

Epidemiology shows that it is very difficult for physicians, regardless of their specialty, not to encounter patients with obesity in their clinical practice.

## Energy homeostasis

Human beings can regulate the amount of adipose tissue possessed in the long term using homeostatic systems that modify caloric consumption and food intake based on their own energy reserves [27].

The brain and peripheral organs communicate intensely and bidirectionally to regulate the energy balance. In the brain, the hypothalamus is the main target for neuronal, hormonal, or food-derived chemical signals that activate neural circuits with orexigenic or anorexigenic functions [28].

A reduction in fat mass activates biological systems that cause a reduction in metabolism and an increase in hunger [29]. On the contrary, an increase in food intake leads to an increase in energy consumption and a sense of satiety [29]. These two pathways, together create a homeostatic energy system, and are intensely influenced by genetic, environmental, and behavioural factors.

In humans, the mechanism that is supposed to protect against excessive fat mass expansion appears to be less efficient than the system that regulate energy balance in case of reduced reserves. The reasons why this occurs have not yet been clarified [30]. Obesity generally occurs when a genetically predisposed individual lives in an obesogenic environment for a long period.

Environmental factors are probably the most influential elements of the epidemic weight gain documented in the last 30 years [31, 32]. Several changes that have affected the labour market as well as personal life, and leisure, have led to a sedentary lifestyle. This, combined with the easy availability of highly processed food creates the conditions for weight gain. In addition, people living in high-income countries are bombarded with advertisements touting cheap food and drinks that should allow the achievement the hypothetical happiness in everyday life. Despite this, among individuals exposed to the same environmental conditions, there is a notable difference in FM suggesting a complex interaction between individual characteristics and environment. Evidence for relevant genetic contributions to body weight comes from family, twin, and adoption studies [33–36], and both human and animal models [33–37].

Genetics can contribute in many ways to generating and maintaining obesity [38–40]. Genetic mutations affecting some specific key proteins of energy homeostasis have been reported [41]. However, these mutations account for a minority of obesity cases. In the vast majority of cases, the individual propensity derives from a set of genes which individually give only a modest contribution, but all together determine an important individual predisposition [37–40]. Large-scale human genome-wide association studies (GWAS) have identified > 500 genetic loci statistically associated with obesity or increased WHR. Although it is not clear what is the function of these identified loci and, more importantly, to what molecular mechanisms the stronger predisposition of some populations compared with others is due, it seems clear that given equal environmental exposure, genetics plays a fundamental role in defining who will be affected by obesity and who among these individuals will more easily develop the common complications [39].

In experimental rat models, destruction of the ventral hypothalamic portion rapidly leads to an overfeeding compulsion in animals, making them develop obesity in a short time [42]. In contrast, stimulation of the same areas leads to a reduction in food craving and consequent weight loss [43]. After these pioneering works, other hypothalamic centres have been identified [44]. These centres are correlated in some way with different moments of eating, such as the phase of food seeking, the moment in which the food is introduced into the body, and with the feeling of satiety [28]. Moreover, studies on the neuronal circuits that trigger feeling of satiety and hunger have shown how a high fat diet (HFD) alters the functioning of some of these circuits and causes the patient to continue consuming unhealthy foods over time [45]. These data show that difficulty in changing diet may also have neural causes and that obesity activates a self-reinforcing harmful cycle [45]. A HFD maintained for a sufficiently long period of time may also induce anatomic changes in the hypothalamus, mainly due to local inflammatory processes [46]. These processes, which have been widely demonstrated in animal models [29, 46–51] and that have been proved to occur in humans as well [47], may be the cause of the difficulty in reducing food intake and may be an organic reason for the maintenance of obesity [29, 46–50].

In human and animal models, some studies seem to show that maternal obesity and maternal nutrition have an impact on the future health of the offspring [52–55]. HFD even during breastfeeding alters hypothalamic melanocortin circuitry, leading to malformation of neuronal projections [55]. The development of the hypothalamus appears to be modulated by the maternal diet, which affect its microscopic anatomy and its function, leading to changes in offspring energetic homeostasis [55]. If a high-fat diet depends on environmental and individual behaviour, it should be emphasized that not all individuals respond to this diet in the same way. Genetics likely plays an important role in determining the magnitude of the inflammatory response to environmental stimuli, although this aspect is not yet well-understood [56, 57].

Summarizing and simplifying the neurohumoral system that regulates energy balance and reserves, we can say that leptin, ghrelin, insulin, and various types of nutrients, especially fatty acids, bind to receptors on first-order neurons located in the nucleus arcuatus of the hypothalamus [49, 58]. These cells are connected by a neuronal network to second-order neurons located in the paraventricular nucleus, which has an anorexigenic function, and the lateral hypothalamic area which, in contrast, has a predominantly orexigenic function [49, 58].

Two orexigenic neuropeptides, neuropeptide Y (NPY) and agouti-related protein (AgRP), are produced by first-order neurons. They stimulate second-order neurons in the lateral hypothalamic area to produce orexin (ORX) and melanin-concentrating hormone (MCH), and inhibit second-order neurons in the paraventricular nucleus [49, 58] to produce oxytocin (OT), thyrotropin-releasing hormone (TRH), and corticotropin-releasing hormone (CRH).

In contrast, the anorexigenic neuropeptides proopio-melanocortin (POM) and cocaine- and amphetamine-regulated transcript (CART), which are produced by first-order neurons, have an opposite effect, i.e., they stimulate the paraventricular nucleus, area that has an anorexigenic effect on behaviour, and inhibit second-order neurons located in the lateral hypothalamic area which on the contrary produce an orexigenic effect [49, 58].

Other extra-hypothalamic circuits are involved in feeding control [49, 58]. The mesolimbic reward system, the nucleus accumbens, the nucleus tractus solitarius, the nucleus parabrachial, the ventral cortex, the tegmental cortex, and other sites are also involved in different ways in the process of food intake regulation [58]. Reward-related brain regions are deeply involved in excess weight gain. They are stimulated by the sight of desired foods and, through unconscious processes of learning/conditioning mechanisms, promote further craving and pursuit of these foods regardless of hunger feeling [59].

In addition to a neuronal system, energy homeostasis is also controlled by a circulating humoral system whose main element is leptin [60]. The two systems are closely linked. Leptin is a 16 kilodalton protein (167 amino-acids) that is produced primarily by white adipose tissue and mediates numerous functions in the body [60]. The gene encoding leptin (*Ob*) is expressed not only in white adipose tissue but also in the stomach, placenta, and mammary glands [60]. Serum leptin levels are positively correlated with fat mass [60]. Leptin increases after the meal and decreases during fasting, which suggests a direct role in informing the central nervous system about the amount of fat available in the stores and the energy balance, suppressing the feeling hunger and promoting the feeling of satiety [61]. Leptin is released into the bloodstream, where it can be detected both freely and bound to the soluble form of its receptor [62]. Leptin also acts by binding to specific receptors in the central nervous system by altering the expression of various neuropeptides [63]. Since leptin is produced mainly in adipose tissue and its preferred target is in the central nervous system, there must be a system through which it passes the blood–brain barrier. It is likely that entry into the central nervous system is mediated by an active system that can be saturated, which explain at least in part, the phenomenon of leptin resistance [64]. Although regulation of fat stores is probably its main function, leptin also has other functions and plays an important role in lactation, regulates bone density and immune system functions, mediates pathophysiological processes activated in pathological conditions as diabetes and hypertriglyceridemia [63, 65]. In fact, leptin receptors are also expressed in many peripheral tissues, such as lung, kidney, liver, pancreas, adrenal glands, ovaries, hematopoietic stem cells and skeletal muscle [60]. Alteration of leptin structure, an inability to bind to its specific receptor or leptin resistance can lead to obesity through complex and not fully understood mechanisms [61]. The discovery of leptin [66], adiponectin [67], ghrelin [68] and many other hormones and cytokines [69] produced by adipose tissue, gives this tissue a role that goes far beyond energy storage, and leads to identify it as the largest endocrine organ [70]. A complete description of all hormones and cytokines and their functions as well as all neuronal circuits and cell types involved in energy homeostasis, although fascinating, is beyond the scope of this text [28, 42–44, 63, 69, 70].

### Take-home messages

The hypothalamus (the main control centre of energetic homeostasis), the mesolimbic system (involved in the reward network), and the prefrontal cortex (the centre responsible for executive control) are closely connected to each other. They regulate food intake in response to environmental stimuli and/or bodily needs [44, 45, 59]. This neuro-endocrine central system constantly receives information from the periphery about the amount of adipose tissue present and about energy balance [49]. The neuro-endocrine system that regulates energy homeostasis also includes circulating mediators, the main one being leptin.

Neurons and glial cells in the hypothalamus jointly regulate various metabolic functions in response to information coming from the periphery, from adipose tissue, and the gut [71].

Hypothalamic inflammation could be the primary mechanism leading to unwanted weight loss or obesity [50]. Obesity creates a self-reinforcing harmful cycle [45].

## Causes of obesity

In general, patient management includes a diagnostic phase, with the collection of anamnestic data and objective information and a subsequent treatment strategy proposal. Unfortunately, most of these steps are often overlooked or sometimes skipped in the management of patients with obesity.

The diagnosis of obesity often coincides with the finding of a BMI of 30 kg/m^2^ or more. The search for modifiable causes and the presence of conditions favouring the development or maintenance of obesity are usually omitted. Indeed, when physicians frequently focus on the management of obesity, they directly propose to the patient a strategy aimed at reducing the adipose tissue or the consequences of their obesity.

In clinical practice, it must be considered that obesity is a multifactorial disease. In simple terms, it is a chronic disease that occurs when the amount of calories ingested exceeds the amount consumed. Although the amount of calories supplied depends on the quantity and quality of food ingested, and the amount of those consumed depends primarily on the level of the physical activity (apart from basal metabolic rate), many factors can influence both calorie intake and expenditure.

**Fig. 1:**
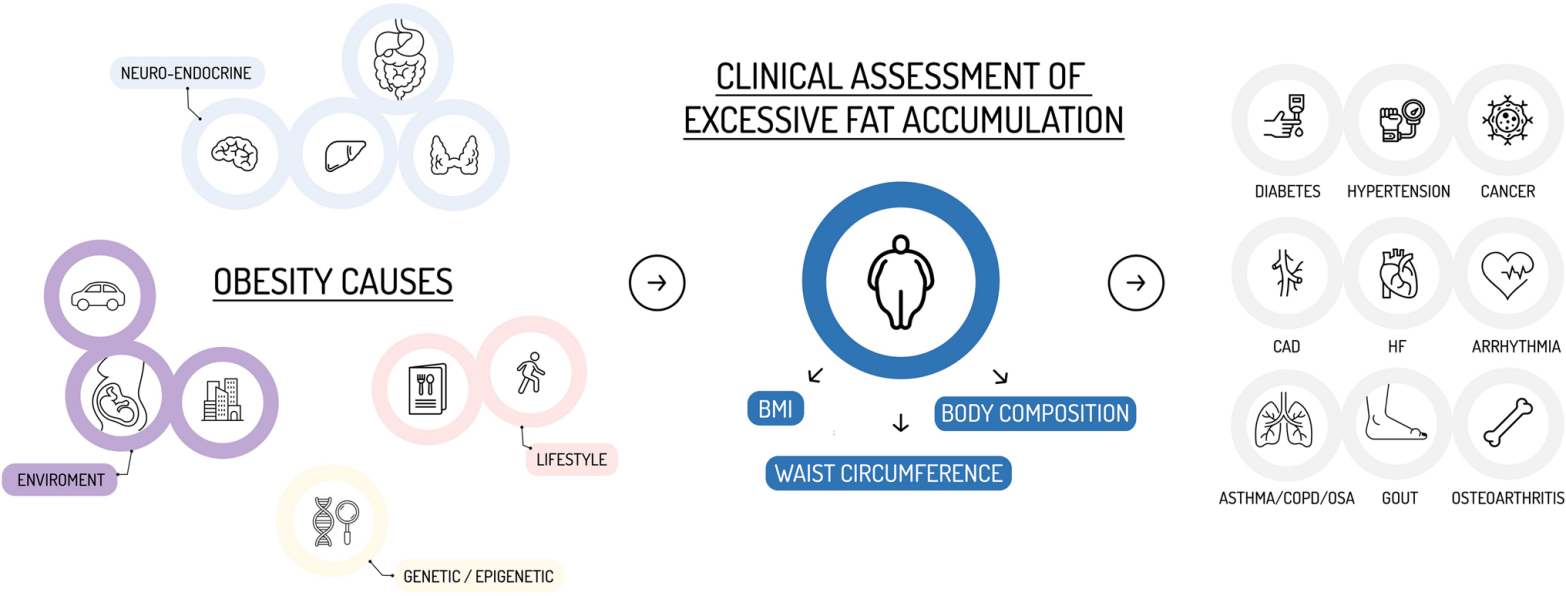
Causes, assessment, and consequences of obesity

Individual factors (genetic [72, 73], epigenetic [73, 74], immunological [75], endocrinological [76], pharmacological [77] or behavioural [78]) and environmental factors [79] contribute in different ways in each person to the development of a chronic caloric excess that generates and maintains the excess adipose tissue (Fig. 1) [77, 80].

The clinical conditions that can produce or maintain an abnormal amount of adipose tissue have been summarize by van der Valk et al. [77] into six groups. These include genetic or syndromic, hypothalamic, endocrine, pharmacological, mental, or lifestyles-related conditions. It is not easy for a cardiologist to know and remember all these clinical conditions, but it is much more difficult to understand the importance and relative weight that each factor has in the single patient. Sometimes, a medical condition is likely to be a cause, other times the same condition can be a consequence of obesity, such as hypothyroidism and hypogonadism [77]. For these reasons, a multidisciplinary approach is mandatory. A working team should include physicians specialized in obesity (obesiologists, cardiologists, pneumologists and bariatric surgeons), dietitians, clinical psychologists, exercise trainers, nurse trainers, social workers.

A mono-disciplinary approach is less effective [81].

Throughout the diagnostic assessment of patients with obesity, every element that is known to potentially contributes to produce or to maintain abnormal fat tissue should be systematically investigated. Genetics, endocrine diseases, medications, prenatal dietary influences of the mother and postnatal dietary influences of both mother and patient, unhealthy diet, low physical activity, a behaviour with unhealthy habits, and the diet culture are all factors that contribute to the genesis of the increase in fat mass. All the elements mentioned above are intertwined with each other, sometimes being obesity cause, sometimes the consequence. In the individual, to deal with this complexity, it is necessary to search, identify, and modify the single thread of the skein through a multidisciplinary approach. The patient should receive concrete tools, such as drugs, but this might not be enough. They must become aware of the scenario in which they move, the elements at play, and the ways to impact on them.

### Take-home message

The cardiologist must become aware that obesity is a chronic and complex disease [2].

The evaluation of a patient with obesity must include the search for causes or favouring conditions [77]. A multidisciplinary team should perform the complex diagnostic assessment [81–85].

If the cardiologist is not a part of a multidisciplinary team, he should advise the patient to contact one by providing the relevant contact during the visit.

## Health consequences of obesity

Numerous studies highlight that excess body weight is a major risk factor for mortality and morbidity [25]. At least 4.0 million deaths are thought to be attributable to obesity each year worldwide [25]. Obesity is a medical problem [81]. It is not simply a phenotype but itself a disease in its own right [86, 87], intricately linked with many other diseases and risk factor [25].

However, a high BMI is not necessarily associated with risk factors for other diseases, organ damage, or comorbidities. In addition, obesity-related psychological symptoms or functional limitations may result from a specific patient predisposition or the duration of obesity persistence. For this reason, it is vital to classify obesity not only based on weight but also on the previously mentioned factors. [88]. The Edmonton Obesity Staging System (EOSS) is a frequently used classification to better describe the patient and to better identify the presence of obesity-related risk factors, physical symptoms, psychological symptoms, functional limitations, or comorbidities [88]. It aids clinical decision-making. An EOSS stage 3 has been found to predict clinical outcomes [89, 90]. Among adults with overweight or obesity, EOSS is a more accurate predictor of polypharmacy and health service use than the BMI [90]. Furthermore, a higher perioperative surgical risk in subjects undergoing bariatric surgery with similar BMI has been found in patients with higher EOSS stages [91]. In addition, at the individual level, a clinical staging such as EOSS predicts mortality better than BMI [92]. Table 4 reports a simplified version of EOSS [88, 90, 93].

**Table 4:** Schematic report of the Edmonton Obesity staging system

Area	Stage 0	Stage 1	Stage 2	Stage 3	Stage 4
Clinical	No risk factor	Subclinical risk factor	Established disease	Severe disease	End stage disease
Mental	No risk factor	Subclinical risk factor	Established disease	Severe disease	End stage disease
Functional	No risk factor	Subclinical risk factor	Established disease	Severe disease	End stage disease

A simplified version of Edmonton Obesity Staging System [85, 87, 90]

Other clinical staging systems have also been proposed to be added to the anthropometric evaluations to better define the patient's health needs and help clinicians in their decisions making, for example, the King's System [94], and the Cardiometabolic Staging System [95]. The latter is focused on the attempt to identify individual cardiovascular risk.

**Fig. 2:**
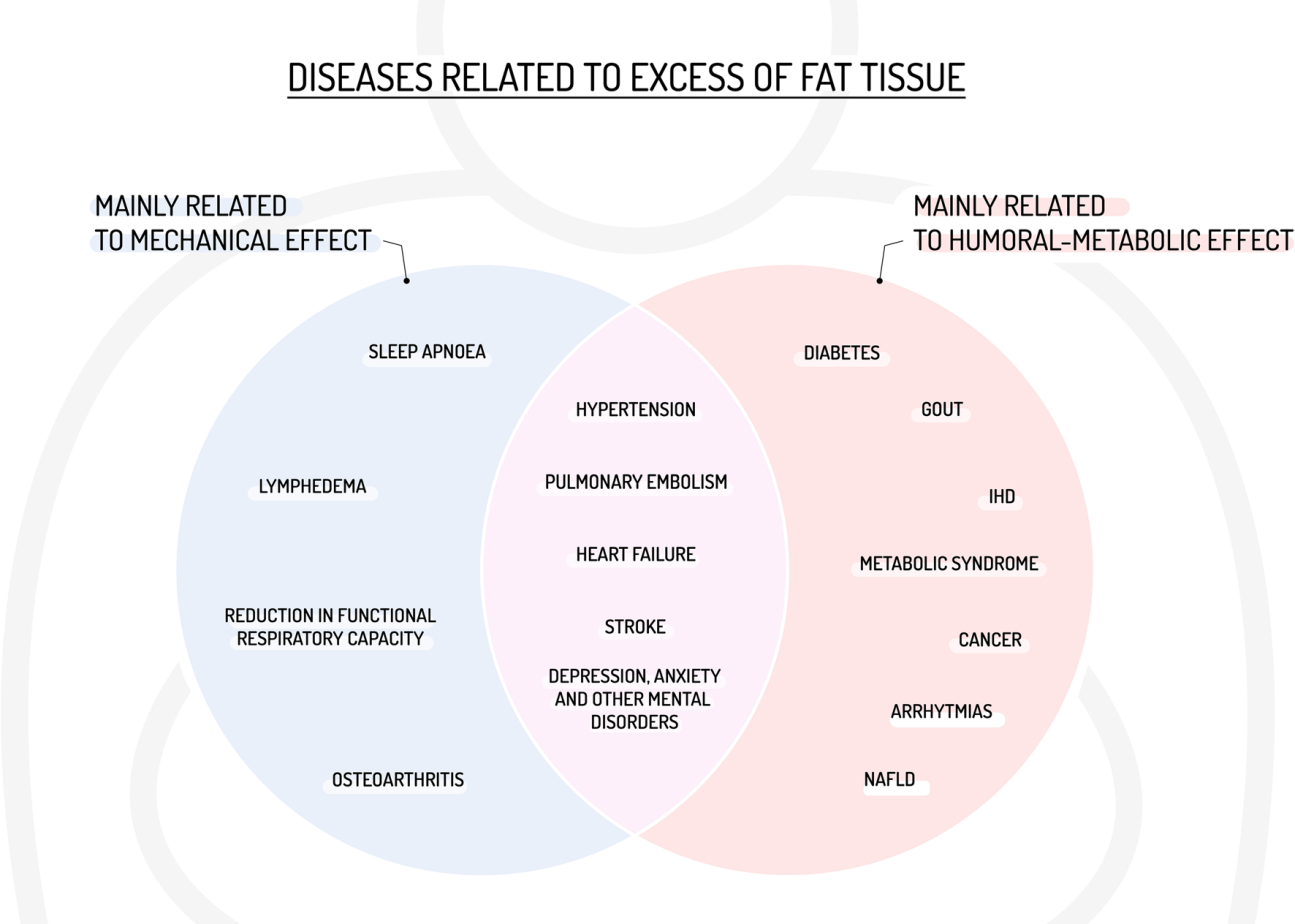
Dual consequences of obesity: mechanical and humoral-metabolic effects

Table 5 summarizes most of diseases related to excess of fat tissue. They could be divided in a simplistic way in two broad categories. The first comprises all clinical situations correlated with the state of chronic inflammation resulting from the increase in adipose tissue, especially visceral, and circulating mediators release, and the second includes the consequences of the mechanical effects of increased body weight and body shape changes (Fig. 2).

**Table 5:** Obesity-related disease

Disease	References
Asthma	[93, 94, 96, 97]
COPD	[93, 95, 96, 98]
OSA	[96, 97, 99, 100]
IHD	[98, 99, 101, 102]
Hypertension	[100, 101, 103, 104]
Ischemic Stroke	[21]
Metabolic Disease	[100, 102, 103, 105]
Gallbladder	[103, 106]
Metabolic Syndrome	[3, 104, 105, 107, 108]
Diabetes	[106, 109]
Dyslipidemia	[100, 103]
Gout	[107, 110]
Non-alcoholic fatty liver disease	[108, 111]
Venous thromboembolic disease,	[109, 112]
Pulmonary embolism	[110, 113]
Heart failure	[111, 112, 114, 115]
Arrhythmias	[113, 114, 116, 117]
Cancer	[115, 116, 118, 119]
Colon	[117, 118, 120, 121]
Kidney	[117, 119, 120, 122, 123]
Oesophagus	[117, 121, 120, 124]
Endometrium	[117, 120, 122, 123, 125, 126]
Postmenopausal breast	[117, 120, 122, 123, 125, 126]
Liver	[124–126, 127-129]
Pancreas	[127, 128, 130, 131]
Thyroid	[129, 132]
Osteoarthritis	[130, 133]
Depression, anxiety, and other mental disorders	[131–134, 136]

CVD, cardiovascular disease; COPD, Chronic Obstructive Pulmonary Disease; IHD, Ischaemic Heart Disease; OSA, Obstructive Sleep Apnoea

### Take home message

Obesity is a medical problem. Patients with similar BMI may have different metabolic conditions, different fit status, and different cardiovascular risk. The EOSS can assist in better understanding the patient health status, and the risk of morbidity or death.

## Obesity paradox

Although epidemiological studies have shown that the presence of obesity is associated with numerous diseases, as reported in Table 5, for some specific diseases, a better outcome has been reported in those overweight or with first-degree obesity (BMI ≥ 30 kg/m^2^ and < 35 kg/m^2^), compared to those who had a BMI < 25 kg/m^2^ [137]. Since the first identification of this counterintuitive finding, numerous similar studies have been published in different fields, such as cardiovascular diseases [138], cancer [139], diabetes [140], respiratory diseases [141] and in other clinical contexts [142]. A recent large cohort study including patients with stable coronary heart disease has found a U‐shaped relationship between BMI and cardiovascular outcomes, with the lowest cardiovascular risk in patients with a BMI of 27 kg/m^2^, which are usually classified as overweight patients [143].

This topic has also been examined through rigorous meta-analyses [144–149] but its interpretation remains not yet clear. Possible explanations range from the real existence of the phenomenon [150], to the presence of various population selection biases [149–152], or the lack of discriminatory power of BMI to differentiate between body fat and lean mass [153].

An interesting clarification of this controversy could come from the distinction between metabolically healthy and metabolically unhealthy obesity, probably considering the former as a transitional phenotype towards the latter [154]. The distinction between these two forms of obesity is not easy. The presence of normal glucose metabolism, normal insulin sensitivity, no systemic arterial hypertension, no drug treatment for dyslipidaemia, diabetes, or hypertension, and no cardiovascular disease manifestation can be used in daily clinical practice to distinguish the two forms of obesity [154]. However, other aspects, such as, for example, the alteration in the secretion of the various cytokines produced by the adipose tissue or the presence of a chronic inflammatory state, if present, are not easy to identify. Of note, a study on heart failure (HF) patients recently showed no evidence of obesity paradox in terms of mortality when using an anthropometric measure different from BMI: the waist-to-height ratio [155]. Overall, to identify obesity risks, an index more efficacious in detecting greater adiposity than the BMI should be used, both in clinical studies, and clinical practice.

### Take home message

Obesity paradox is a not yet clearly defined phenomenon. Studies that use an obesity index different from BMI are needed to verify the hypothesis of the existence of an obesity paradox phenomenon in cardiovascular diseases.

## Cardiovascular diseases

Cardiologists are deeply involved in the evaluation of obesity and play a pivotal role in this field for several reasons. First, they have a high likelihood of encountering patients with this condition. Second, the cardiovascular risk profile is among the most crucial elements to quantify to determine the additional risk posed by obesity and to assess the extent of clinical benefit that an individual patient can achieve through weight loss [156].

Although the relationship between obesity and cardiovascular disease is very complex due to the presence of many confounding factors, Mendelian randomization studies strongly show a causal relationship [157].

Obesity is linked to many cardiovascular diseases, including stroke [158], venous thromboembolic disease [112], pulmonary embolism [113], HF [114], and arrhythmias, especially sudden cardiac death (SCD) [116] and atrial fibrillation (AF) [117]. Furthermore, it causes specific issues in key topics for cardiologic patient management, such as differences in specificity and sensitivity in non-invasive and invasive diagnostic tools [159], changes in platelet reactivity [160, 161], a different short- and long-term outcome after percutaneous coronary intervention PCI [162–164] or cardiac surgery [159].

### Coronary artery disease and obesity

Patients affected by obesity show some differences in disease pathophysiology, sensitivity, and specificity of diagnostic tools, and in outcomes when compared with normal weight patients [159].

Overweight and obesity are associated with elevated coronary artery disease (CAD) risk at least in part independent of blood pressure and cholesterol levels [157, 165].

The link between increased cardiovascular risk and obesity can be explained by multiple mechanisms [166], such as increased incidence of hypertension [103, 104], type II diabetes [109] and insulin resistance [167], hypercholesterolemia [103], systemic inflammation [168], endothelial dysfunction [169] and increased platelet aggregability [161].

Weight loss improves systemic inflammation, endothelial dysfunction, and related atherosclerotic risk factors for coronary artery disease, such as hypertension, metabolic syndrome, and diabetes [159].

#### CAD diagnostic assessment

Electrocardiography, stress test including ergometric ECG test, nuclear medicine tests, stress echocardiography, or MRI, coronary artery calcium (CAC) scan screening, CT, and coronary angiography are CAD evaluation methods that have some specific limitations in patient living with obesity.

The electrocardiographic criteria of normality in patients with obesity may be different from those for patients with normal weight, and several electrocardiographic changes are associated with obesity resulting in lower ECG sensitivity and specificity [159].

In patients with obesity, treadmill or cycle-ergometer ECG stress tests have some limitations. The patients could not achieve the maximal theoretical heart rate for orthopaedic limitations or some degree of chronotropic incompetence [170]. Pulmonary dysfunction has been described and can limit the attainable level of work [171].

Single-photon emission CT, stress echocardiography, and cardiac CT coronary angiography all have limitations for patients with obesity [159]. The sensitivity and specificity of these techniques may decrease compared to those of normal size patients [159]. Conversely, stress cardiac MRI and rubidium PET myocardial perfusion imaging [172] may offer better results. Therefore, PET rubidium, if available, should be the nuclear imaging technique of choice for patients with obesity [172].

As far as invasive examinations are concerned, the major problems concern the difficulty in vascular access and radioscopic views. In some cases, the angiography tables may have limitations in terms of weight they can support.

### Heart failure

Obesity is a major risk factor for HF development [115, 173]. When an obese patient has HF in the absence of other factors that justify it, we can refer to it as obesity cardiomyopathy [174]. Obesity causes several functional and structural changes that may lead to HF. Among these we can mention arterial hypertension, respiratory dysfunction, the increase in circulating blood volume and cardiac output, vascular remodelling, sleep apnoea syndrome, the presence of chronic systemic inflammation, abnormal secretion by the adipose tissue of numerous chemical mediators [174, 175].

A substantial weight loss positively modifies many hemodynamic parameters involved in HF development and progression. Among them we can find cardiac output, central blood volume, stroke volume and stroke work, systemic and pulmonary pressure [176].

The reduction in systemic blood pressure is probably the most relevant hemodynamic element that is observed with weight loss and may improve cardiovascular function [176].

### Sudden cardiac death

Several studies identified a close relationship between obesity and the risk of SCD [177, 178]. The pathophysiological mechanisms underlying this association are numerous. Among them there is a greater number of traditional risk factors for cardiovascular diseases, the presence of both electrical and structural ventricular remodelling, the presence of chronic inflammation and of an autonomic nervous system imbalance [178].

It is not known whether weight reduction is able to modify the incidence of sudden death in this category of patients. In a Swedish study that followed 2010 patients after bariatric surgery [179] for about 11 years, a statistically non-significant increase in sudden death was observed compared to the control group, whereas a reduction in global mortality was observed in the surgery group. Further studies on this topic are needed to understand the relationship between obesity and sudden death and the consequences of weight loss.

### Atrial fibrillation

Increased BMI is an established risk factor for the development of AF [180]. These data have already been highlighted many years ago by monitoring the postoperative period after cardiac surgery [181].

Furthermore, weight reduction corresponds to a reduced risk of developing AF [182], while the persistence of obesity favours the transition from paroxysmal to persistent atrial fibrillation [183].

A 1 kg/m^2^ increase in BMI was associated with a 4.7% increase in the likelihood of experiencing an episode of AF [184]. Conversely, a reduction of 1 kg/m^2^ was associated with a 7% reduction in AF incidence [182].

Although the concept of obesity paradox in which a reduction in mortality is observed in overweight or moderately obese patients compared to patients of normal weight, has been reported in AF patients, a study which set out to analyse non-healthy and healthy obesity effect showed an increase in the incidence of this arrhythmia also in the latter group of patients [185].

Obesity can induce AF through several mechanisms. First, it contributes to several hemodynamic changes, diseases, or cardiovascular disease risk factors, such as arterial hypertension, type II diabetes, sleep apnoea, volume overload, left ventricular remodelling, which in turn may contribute to AF occurrence. Furthermore, obesity also determines a series of histological and biochemical changes closely related to arrhythmic risk. The increase in epicardial fat, along with the increase in the production of inflammatory and profibrotic mediators, which have both endocrine and paracrine effects, may explain the higher risk of AF among patients with obesity [180]. Of note obesity may also affect anticoagulant drug pharmacokinetics and pharmacodynamics and in patients with severe obesity the anticoagulant treatment must be individualized [142].

### Take home message

Ischemic heart disease, HF, arrythmias are more frequent in patients with obesity than in the general population. Patients affected by obesity exhibit some differences in cardiovascular pathophysiology, sensitivity, and specificity of diagnostic tests, as well as outcomes when compared to normal-weight patients.

## Executive summary

Approaching a patient with obesity is not easy for many reasons. First, the patient may not be ready to face the problem; he/she may have internalized the stigma to which he/she has certainly been subjected. He/she may feel that he/she does not have the strength to face the obstacles that stand in the way of losing weight. Furthermore, BMI is not adequate to measure body composition neither to identify which patients are most prone to obesity-related complications. Different tools and methods are needed to correctly classify patients and support clinical decisions. In addition to BMI the WC, WHR, and WHtR should be used for clinical purposes (Table 1). Clinical staging systems should also be used to better identify the global health status of the patients and the risk of death or disease.

Very often these patients have experienced numerous failures in weight loss in their past or suffer from of real eating disorders. International guidelines [186] propose a systematic approach through the application of a method that can be summarized in “5As”: Ask permission, Assess their story, Advice on management, Agree on goals, Assist with drivers and barriers.

The cardiologist has a high probability of encountering patients who live with obesity in their clinical practice and, therefore, must become a privileged interlocutor for these patients.

It is necessary to recognize that obesity is not the consequence of laziness or weakness of character and become aware that factors that lead to obesity are almost never controllable by the patient without a professional support.

Cardiologists must develop the knowledge necessary to relate to patients affected by obesity and learn to distinguish which patients are at higher risk of poorer outcomes. In routine practice the assessment of a patient with obesity, as defined with BMI measurement, or with a suspicion of excess body fat, must include additional measurements to estimate fat distribution (WC, WHR, WHtR). Table 6 reports the main laboratory tests to consider in the evaluation of patients with obesity.

**Table 6:** Laboratory tests in the evaluation of patients with obesity

Renal function tests (creatinine, eGFR), electrolytes (sodium, potassium, magnesium, calcium)
HbA1c
Total cholesterol, HDL- and LDL-cholesterol, triglycerides
Alanine aminotransferase and Aspartate aminotransferase
Complete blood cell count
Thyroid stimulating hormone
Uric acid
Assessment of iron (total iron binding capacity; % saturation, serum ferritin, serum iron)
Vitamins B12 and D levels
Urinalysis
Urine for micro-proteinuria
Women with suspect of polycystic ovary syndrome
LH, FSH, total testosterone, DHEAS, prolactin and 17 hydroxyprogesterone level

LH, luteinizing hormone; FSH, follicle stimulating hormone; DHEAS, dehydroepiandrosterone

Since the disease is very complex, a multidisciplinary approach is essential in both the diagnostic and therapeutic phases. The latter must include curative diet therapy, the prescription of drugs with documented efficacy, psychological and social support, adequate physical activity and, if necessary, bariatric surgery.

## Conclusion

Obesity is a complex, chronic, and relapsing disease [2]. Due to a rapid spread of an obesogenic environment and a genetic predisposition that affects a large part of the human population, obesity has become an epidemic and is spreading in all countries of the world with a growing prevalence [22–25].

Patients living with obesity have a high probability of developing risk factors for cardiovascular diseases, such as systemic arterial hypertension, type II diabetes mellitus, sleep apnoea, and dyslipidaemia [26]. The risk of developing ischaemic heart disease, sudden death, heart failure, and atrial fibrillation is also high [159, 187].

In the near future the increase in the prevalence of the disease will also lead to an increase in its consequences, but it is also envisaged a significant increase in the weapons at our disposal. The pharmacological treatment of obesity is currently limited to a small number of drugs but a considerable increase in available products in the coming years is expected thanks to the increasing knowledge of the obesity physiopathology and the mechanisms of the energy homeostasis regulation [188].

The possibility of halting and reversing the spread of obesity will probably depend on our ability to propose a systemic view of the disease and a close collaboration among all the professional figures involved. A single discipline approach will certainly be less effective.

## Strength and limits

"This paper represents a scientific statement from the largest scientific society of Italian cardiologists. Therefore, it can be an important tool for increasing awareness about obesity among cardiologists, improving the quality of knowledge, and consequently, the quality of care. The main limitation stems from the unsystematic approach to the analysis of the literature. However, this element is not required, given the very nature of the paper as a scientific statement."

## What is already known on this subject?

Patients with obesity consult doctors from numerous specializations, and among these, cardiologists are certainly some of the most frequently visited due to comorbidities and the increased risk of cardiovascular issues. Despite this, it is not always easy for doctors who do not directly deal with obesity to understand the nature of obesity as a chronic pathology. This can lead to difficulties in implementing appropriate actions without resorting to simplistic or even stigmatizing clinical approaches and therapeutic proposals.

## What this study adds?

This paper reviews the literature to provide cardiologists with a theoretical and practical framework for offering adequate care to patients with obesity. This care is approached from a global perspective, with specific reference to cardiovascular pathology.

## Data Availability

Not applicable.
